# Associations of Texture Features of Proton Density Fat Fraction Maps between Lumbar Vertebral Bone Marrow and Paraspinal Musculature

**DOI:** 10.3390/biomedicines10092075

**Published:** 2022-08-25

**Authors:** Yannik Leonhardt, Michael Dieckmeyer, Florian Zoffl, Georg C. Feuerriegel, Nico Sollmann, Daniela Junker, Tobias Greve, Christina Holzapfel, Hans Hauner, Karupppasamy Subburaj, Jan S. Kirschke, Dimitrios C. Karampinos, Claus Zimmer, Marcus R. Makowski, Thomas Baum, Egon Burian

**Affiliations:** 1 Department of Diagnostic and Interventional Radiology, School of Medicine, Klinikum rechts der Isar, Technical University of Munich, 81675 Munich, Germany; 2 Department of Diagnostic and Interventional Neuroradiology, School of Medicine, Klinikum rechts der Isar, Technical University of Munich, 81675 Munich, Germany; 3 TUM-Neuroimaging Center, Klinikum rechts der Isar, Technical University of Munich, 81675 Munich, Germany; 4 Department of Diagnostic and Interventional Radiology, University Hospital Ulm, 89070 Ulm, Germany; 5 Department of Neurosurgery, University Hospital, Ludwig-Maximilians-University (LMU) Munich, 81377 Munich, Germany; 6 Institute of Nutritional Medicine, School of Medicine, Klinikum rechts der Isar, Technical University of Munich, 81675 Munich, Germany; 7 Sobey School of Business, Saint Mary’s University, Halifax, NS B3H 3C3, Canada

**Keywords:** magnetic resonance imaging, proton density fat fraction, texture analysis, quantitative imaging, muscle composition

## Abstract

Chemical shift encoding-based water–fat MRI (CSE-MRI)-derived proton density fat fraction (PDFF) has been used for non-invasive assessment of regional body fat distributions. More recently, texture analysis (TA) has been proposed to reveal even more detailed information about the vertebral or muscular composition beyond PDFF. The aim of this study was to investigate associations between vertebral bone marrow and paraspinal muscle texture features derived from CSE-MRI-based PDFF maps in a cohort of healthy subjects. In this study, 44 healthy subjects (13 males, 55 ± 30 years; 31 females, 39 ± 17 years) underwent 3T MRI including a six-echo three-dimensional (3D) spoiled gradient echo sequence used for CSE-MRI at the lumbar spine and the paraspinal musculature. The erector spinae muscles (ES), the psoas muscles (PS), and the vertebral bodies L1-4 (LS) were manually segmented. Mean PDFF values and texture features were extracted for each compartment. Features were compared between males and females using logistic regression analysis adjusted for age and body mass index (BMI). All texture features of ES except for Sum Average were significantly (*p* < 0.05) different between men and women. The three global texture features (Variance, Skewness, Kurtosis) for PS as well as LS showed a significant difference between male and female subjects (*p* < 0.05). Mean PDFF measured in PS and ES was significantly higher in females, but no difference was found for the vertebral bone marrow’s PDFF. Partial correlation analysis between the texture features of the spine and the paraspinal muscles revealed a highly significant correlation for Variance(global) (r = 0.61 for ES, r = 0.62 for PS; *p* < 0.001 respectively). Texture analysis using PDFF maps based on CSE-MRI revealed differences between healthy male and female subjects. Global texture features in the lumbar vertebral bone marrow allowed for differentiation between men and women, when the overall PDFF was not significantly different, indicating that PDFF maps may contain detailed and subtle textural information beyond fat fraction. The observed significant correlation of Variance(global) suggests a metabolic interrelationship between vertebral bone marrow and the paraspinal muscles.

## 1. Introduction

The vertebral body consists of the cortical bone, the trabecular bone and, interspersed in the cavities of it, adipocytes in yellow marrow regions or adipocytes and hematopoietic red blood cells in red marrow regions [1,2]. The composition of the mineralized as well as the non-mineralized parts of the bone is not constant and can change in the context of aging or various metabolic diseases. For instance, osteoporosis is a manifestation of gradual loss of bone mass and deterioration of cortical and trabecular bone microarchitecture, which can lead to increased bone fragility and low-energy fractures of the vertebrae; however, it has also been shown that patients with osteoporosis have significantly increased bone marrow fat fraction (BMFF), which suggests a shift of differentiation of mesenchymal stem cells to adipocytes rather than osteoblasts [3,4]. Other metabolic diseases also manifest in altered BMFF, e.g., among patients with type 2 diabetes mellitus (T2DM) high HbA1c levels were associated with significantly higher BMFF [5].

Similar to changes in bone structure, studies have also shown a change in the composition of paraspinal musculature as a result of various diseases. Specifically, patients with T2DM, but also patients with low back pain, injuries, and neuromuscular diseases have demonstrated measurable changes in the fat content of the paraspinal musculature [6,7,8,9,10]. Post-menopausal women have a significantly higher degree of muscle fat infiltration (MFI), which suggests a concordant change of vertebral and muscle composition in context of the disease [11,12].

For non-invasive assessment of regional body fat distributions, advanced MRI-based techniques have proved to be promising tools, as they enable precise in vivo quantitative evaluation without ionizing radiation, such as magnetic resonance spectroscopy (MRS) and chemical shift encoding-based water–fat MRI (CSE-MRI) [13]. Specifically, CSE-MRI represents a field map-insensitive technique to determine fat and water signal of the measured body compartment. This makes a precise quantification of water–fat compositions possible by determining the proton density fat fraction (PDFF), which has shown to be a reliable and non-invasive approach to extract quantitative information about fat compositions, showing very good concordance with histology or MRS [14,15,16,17]. In a previous study, Sollmann et al. applied this technique and investigated the associations between lumbar vertebral bone marrow and paraspinal muscle fat compositions in pre- and post-menopausal women by measuring the PDFF in the respective compartments; they could show that there are significant correlations between the PDFF of paraspinal muscle and vertebral bone marrow compartments in post-menopausal women [12].

In recent literature however, texture analysis (TA) has been applied to CSE-MRI-based PDFF maps in order to extract even more quantitative information that might be present in the imaging data, especially as mean PDFF does not address the variability in muscular and vertebral structure [18,19]. These studies have demonstrated the feasibility of TA based on CSE-MRI-derived PDFF maps in vertebral bone marrow and in paraspinal muscles, where TA could predict muscle strength based on the pattern of MFI, while specific texture features can be used to describe spatial heterogeneity of vertebral bone marrow in pre- and post-menopausal women, where heterogeneity is higher in post-menopausal women [18,19]. However, there has been no study that investigated the texture features derived from CSE-MRI of the vertebral bone marrow in the lumbar spine in a cohort of healthy males and females, and also the associations between texture features of the vertebral bone marrow and lumbar muscle compartments. Thus, this study aimed to investigate possible associations between vertebral bone marrow and paraspinal muscle texture features derived from CSE-MRI-based PDFF maps in healthy males and females.

## 2. Materials and Methods

### 2.1. Study Population

For this study, 44 volunteers (13 males, 55 ± 30 years; 31 females, 39 ± 17 years) were recruited at the Institute for Nutritional Medicine, Klinikum rechts der Isar, Technical University of Munich, Germany, from a large observational study aiming at evaluating determinants of basal metabolic rate. The study was approved by the local institutional review board (Ethics Commission of the Medical Faculty, Technical University of Munich, Germany; Ethics proposal number 165/16 S; date of approval: 12 May 2016). Inclusion criteria were age of at least 18 years, no history of severe diseases, no surgery within the last three months, no acute physical impairment, and no contraindications for MRI (e.g., incompatible implants). All subjects gave written informed consent prior to study inclusion.

### 2.2. Magnetic Resonance Imaging

All subjects underwent MRI at 3T (Ingenia, Philips Healthcare, Best, The Netherlands). An axial and sagittal six-echo multi-echo three-dimensional (3D) spoiled gradient-echo sequence was applied for chemical shift encoding-based water–fat separation using anterior and posterior coil arrays. The sequences acquired the six echoes in a single repetition time (TR) using non-flyback (bipolar) read-out gradients and covered the lumbar spine region with the following imaging parameters for the axially acquired sequence: TR/echo time (TE)1/ΔTE = 8.2/1.24/1.0 ms, flip angle = 5°, field of view (FOV) = 400 × 300 × 140 mm^3^, acquisition matrix size = 268 × 200, acquisition voxel size = 1.5 × 1.5 × 1.5 mm^3^, SENSE with reduction factor = 2.5 × 1.0, receiver bandwidth = 1413 Hz/pixel, 2 averages, and scan time = 2 min and 1 s. The following parameters were used for the sagittally acquired sequence: TR/TE1/1TE = 11/1.4/1.1 ms, FOV = 220 × 220 × 80 mm^3^, acquisition matrix size = 224 × 224 × 20, acquisition voxel size = 0.98 × 0.98 × 4.00 mm^3^, receiver bandwidth = 1527 Hz/pixel, frequency direction = anterior-posterior (to minimize breathing artifacts), 1 average, and scan time = 1 min and 17 s. A flip angle of 3° was used to minimize T1-bias effects [20].

### 2.3. Segmentation and PDFF Measurements

**Figure 1:**
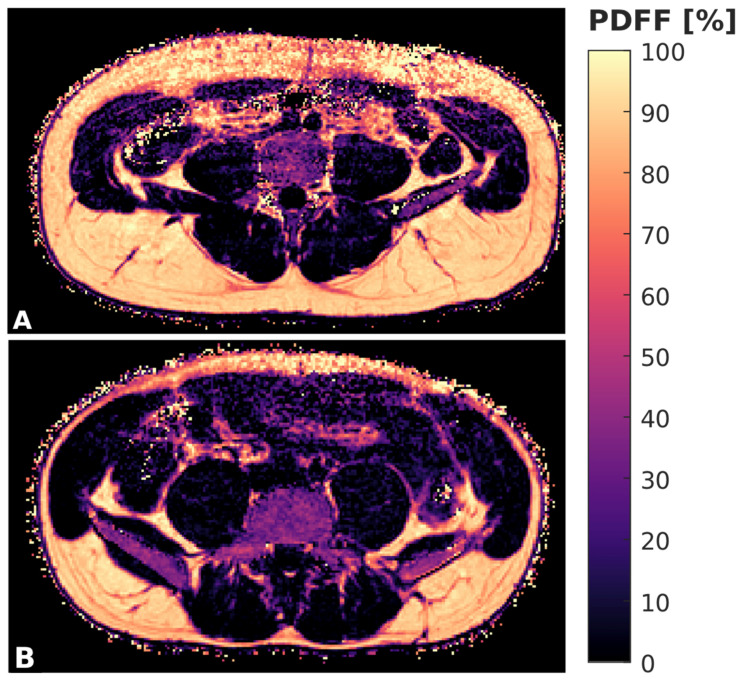
Color-coded, axially acquired PDFF maps derived from CSE-MRI of a 33 year old male (**A**) and 27 year old female (**B**) subject.

The gradient echo imaging data were processed online using the fat quantification routine of the MRI vendor. The routine procedure first performed a phase error correction and then a complex-based water–fat decomposition using a pre-calibrated seven-peak fat spectrum and a single T2* to model the signal variation with TE. The imaging-based PDFF map was computed as the ratio of the fat signal over the sum of fat and water signals (Figure 1).

Segmentations were performed on the axial reformations. All the segmentations were performed by a medical doctor (F.Z.) by outlining the regions of interest (ROIs) on each slice of the PDFF maps using the open-source software MITK (Medical Imaging Interaction Toolkit, German Cancer Research Center, Division of Medical and Biological Informatics, Heidelberg, Germany).

Right and left erector spinae muscles (ES) as well as right and left psoas muscles (PS) were segmented separately from the upper endplate level of L2 to the lower endplate level of L5. Each vertebral body L1-4 was segmented separately. ROIs were placed at the inner contours of the muscles and vertebrae to avoid segmenting subcutaneous fat or surrounding structures. Mean PDFF of each of the four muscles was extracted. For both muscle groups (ES and PS), right and left mean PDFF values were averaged and weighted by the respective muscle volumes to obtain bilateral mean PDFF values (PDFF_ES_, PDFF_PS_). For PDFF of the lumbar spine (LS), measurements from L1-4 were averaged (PDFF_LS_).

### 2.4. Texture Analysis

TA was performed on the PDFF maps of the segmented paraspinal muscles and lumbar vertebrae. The three first-order features Variance(global), Skewness(global), and Kurtosis(global) were extracted. Additionally, eight second-order features were extracted: Energy, Entropy, Contrast, Homogeneity, and Correlation were calculated according to [21], Variance and Sum Average according to [22], and Dissimilarity according to [23]. All texture features were calculated individually for each of the segmented lumbar vertebrae, and each of the four segmented muscles. Texture feature values of vertebral bone marrow were averaged across the four segmented vertebrae (LS). For both muscle groups (ES and PS), values were averaged over both sides, and weighted by the respective muscle volumes.

The first-order features were extracted from intensity histograms. The number of bins used in our analysis, analogous to previous studies, was calculated by taking the median of three different methods, known as Sturges’ method, Scott’s method, and the Freedman–Diaconis method [24,25,26]. Second-order features were extracted using gray level co-occurrence matrix (GLCM) analysis [21]. As a preprocessing step, gray level quantization of the PDFF maps was performed to prevent sparseness by normalizing the image intensities, using 200 equally sized bins and the minimum and maximum gray levels present, corresponding to PDFF values of 0% and 100%, respectively. Entries of the GLCM were obtained by computing the joint probability of two adjacent voxel intensities at a given offset *d =* (*dx*, *dy*, *dz*) and angular directions *θ* = (0, 45, 90, and 135°); *dx*, *dy*, and *dz* denoted the displacement along the *x*-, *y*-, and *z*-axis, respectively. For 3D GLCM analysis, the co-occurrence probabilities of voxel intensities were computed from the 26 neighbors, aligned in the 13 possible angular directions, taking into account discretization length differences. Averaging the texture features computed over the 13 directions ensured rotation invariance. Image processing (including isotropic resampling, uniform gray level quantization, and TA) was performed using MATLAB 2018 (MathWorks Inc., Natick, MA, USA) and a radiomics toolbox (https://github.com/mvallieres/radiomics/, accessed on 4 February 2022) [27,28,29].

### 2.5. Statistical Analysis

Statistical analyses were performed with SPSS 26.0 (SPSS Inc., Chicago, IL, USA) using a two-sided level of significance α = 0.05 for all statistical tests. The Kolmogorov–Smirnov test indicated normally distributed data for the majority of parameters. Mean and standard deviation (SD) of age, BMI, PDFF_ES_, PDFF_PS_, PDFF_LS_, and texture features were calculated for males and females. Mann–Whitney tests were applied to compare male and female subjects in terms of subject characteristics (i.e., age and BMI). Sex-dependent differences of texture features were analyzed using logistic regression models, adjusted for age and BMI. Partial correlation analysis, adjusted for age and BMI, was performed to identify associations between texture features of the lumbar vertebral bone marrow (LS) and paraspinal muscles (ES and PS), respectively.

## 3. Results

### 3.1. Measurements of PDFF and Texture Features

All 44 patients (13 males, 31 females) underwent CSE-MRI. Mean, SD, and *p*-values resulting from Mann–Whitney U tests for age and BMI and from logistic regression analysis (adjusted for age and BMI) for all texture features are displayed in Table 1.

There was a statistically significant difference between males and females in terms of age (males, 54.8 ± 29.5 years; females, 38.7 ± 16.7 years; *p* = 0.043), but not regarding BMI (males, 26.9 ± 4.6 kg/m^2^; females 24.9 ± 4.1 kg/m^2^; *p* = 0.203).

When comparing the results for ES, almost all texture features showed a significant difference between males and females: Variance(global), Kurtosis(global), Energy, and Homogeneity were greater in male subjects while Skewness(global), Contrast, Entropy, Correlation, Variance, and Dissimilarity were greater in female subjects (*p* < 0.05). Only Sum Average was not significantly different between males and females (*p* = 0.853). The PDFF of ES was significantly higher in female subjects with 16.11 ± 9.27% vs. 7.79 ± 6.88% in male subjects (*p* = 0.014).

In the analysis of PS, three features showed a significant difference: Variance(global) was significantly higher in male subjects, while Skewness(global) and Kurtosis(global) were significantly higher in females (*p* < 0.05). All other texture features measured for PS showed no sex-specific differences. PDFF was significantly higher in female subjects with 5.39 ± 4.17% vs. 3.63 ± 5.38% in male subjects (*p* = 0.043).

When analyzing the bone marrow of LS, there were also three features that differed significantly: Variance(global) was significantly higher in male subjects, while Skewness(global) and Kurtosis (global) were significantly higher in females (*p* < 0.05), similar to the results for PS. The PDFF in the lumbar spine, however, was not significantly different in males versus females (33.40 ± 6.77% in males, 33.08 ± 10.30% in females; *p* = 0.917).

### 3.2. Associations between Texture Features of the Bone Marrow and the Muscle Compartments

**Figure 2:**
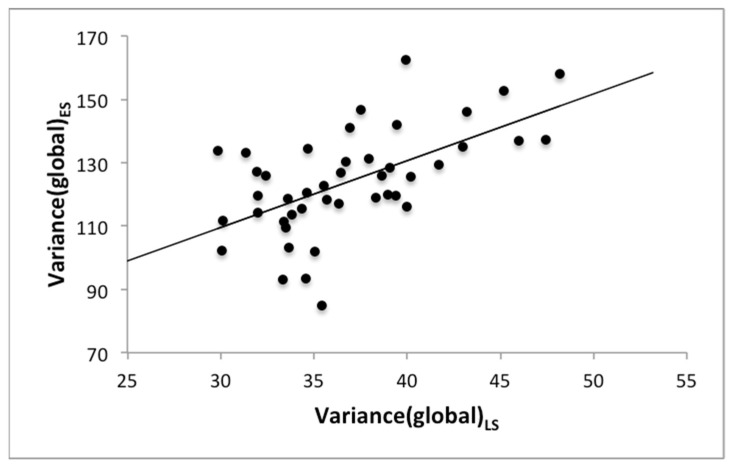
Scatter plot of the measurements of the texture feature Variance(global) for the lumbar spine (LS) and the erector spinae muscle (ES).

In the correlation analysis between the features of ES and LS, there was a high and significant correlation for Variance(global) (r = 0.61, *p* < 0.001; Table 2 and Figure 2). Similarly, there was also a significant correlation for Variance(global) of PS and LS (r = 0.62, *p* < 0.001). All other texture features showed no significant correlation between the vertebral bone marrow and the paraspinal musculature.

## 4. Discussion

In this study, we could demonstrate that texture features of paraspinal musculature and lumbar vertebral bone marrow, extracted from CSE-MRI-derived PDFF maps, show sex-specific differences, even when the overall PDFF in lumbar vertebral bone marrow showed no significant difference between male and female subjects. We also observed significant associations between global texture features in the lumbar vertebral bone marrow and paraspinal musculature.

Previous studies have demonstrated that several diseases can affect the fat composition of vertebral bone marrow and paraspinal muscles [6,7,8,9,11]. In subjects with osteoporosis, for instance, it has been shown that BMFF is significantly higher when compared to healthy subjects [13]. Different techniques have been used to demonstrate this effect, e.g., Griffith et al. used MRS [3], while other researchers applied chemical shift-encoding water–fat separation techniques to demonstrate that the PDFF was greater in osteoporotic than in normal vertebrae [30]. Also, BMFF seems to increase especially in females with increasing age when compared to males, corresponding to the well-known findings that particularly post-menopausal females are prone to osteoporotic vertebral compression fractures [31]. Previous studies have demonstrated that osteoporosis and post-menopausal status in females are also associated with changes of the composition in paraspinal muscles, resulting in an increased MFI [11,12]. While Kim et al. used semi-quantitative measurements by applying three visual scale grades in conventional, qualitative MRI datasets, Sollmann et al. successfully applied PDFF measurements based on CSE-MRI to show this effect [11,12]. While the above-mentioned studies showed the effect of increased MFI and BMFF independently in e.g., osteoporotic cohorts, Sollmann et al. were able to demonstrate significant correlations between the PDFF of paraspinal muscles and the PDFF of vertebral bodies in post-menopausal women, which suggests a close relationship between spatially and functionally associated (i.e., for body movement, spine stabilization, and balance keeping) compartments at the level of the lumbar spine in terms of fat compositions; however, this study focused only on females [12].

In recent literature, TA has been proposed to reveal even more detailed information about the vertebral or muscular fat composition beyond PDFF. In a feasibility study, Burian et al. performed TA of vertebral bone marrow in CSE-MRI-based PDFF maps and demonstrated that certain texture features (Contrast and Dissimilarity) were able to differentiate pre- from post-menopausal women equally well as mean PDFF [18]. Dieckmeyer et al. applied this technique to the paraspinal muscle tissue in 26 healthy males and females and showed that TA of CSE-MRI-based PDFF maps is feasible in paraspinal muscles and predicts muscle strength better than mean PDFF, indicating that muscular function is related to muscle fat distribution [19]. Thus, TA seems to be feasible for both vertebral bone marrow and paraspinal musculature.

According to our data, TA of ES showed significant differences between males and females for all global and all second-order features except for Sum Average. This is in contrast to the features measured for PS, where only the global features showed a significant difference between male and female subjects. These results suggest that the fat distribution and distribution patterns in ES might be more sex-specific than in PS. However, TA of the vertebral bone marrow also revealed sex-specific differences for the global features. This is particularly interesting as the overall PDFF of LS was not significantly different for male and female subjects, whereas PDFF in the muscle compartments was significantly higher in females. These results are in line with those from Dieckmeyer et al., who reported sex-dependent differences in texture features of ES and PS. However, their results also showed significant differences in some second-order features for PS (Energy, Contrast, Entropy, Homogeneity, Variance and Dissimilarity), which seems to be in contrast to our results [19].

In partial correlation analysis, Variance(global) showed significant correlations between the lumbar vertebral bone marrow and both ES and PS. However, the other first-order and all of the second-order features showed no significant correlations. These results are not unexpected, as bone marrow and skeletal musculature are inherently different tissue types; while the overall intra-individual fat content might change concordantly, it is not surprising that in-depth TA revealed that almost all second-order features have no significant correlation. However, Variance(global) seems to be an interesting and potentially useful texture feature, as it showed a high and concordant association between bone marrow and muscles as well as a significant difference between female and male subjects. Further, as stated above, the PDFF in the vertebral bone marrow showed no significant difference between males and females, while Variance(global) showed a significant difference; this might indicate that there is indeed detailed and subtle textural information in PDFF maps, especially in Variance(global), representing the degree of fat distribution heterogeneity, which might not be represented by PDFF measurements without TA.

The present study is not without limitations. First, the comparatively small number of male subjects (n = 13) has to be regarded as a main limitation. The present results have to be interpreted with caution and should be confirmed by follow-up studies with a higher number of subjects. Second, TA was performed on ROIs from manual segmentation enclosing the ES and PS, which were segmented as a whole, thus both intra- and inter-muscular fat contributed to the PDFF distribution and the subsequent TA. Third, we only included healthy subjects in our study. As described above, changes in fat distribution are of particular interest in patients suffering from metabolic diseases such as T2DM. Therefore, future studies should focus on such conditions for exploring the usefulness of PDFF and texture features as well as the interrelationship between the different compartments in a clinical setting.

## 5. Conclusions

Texture analysis using PDFF maps based on chemical shift encoding-based water–fat MRI can distinguish between healthy male and female subjects, particularly when applied to the erector spinae muscles. Global texture features in the lumbar vertebral bone marrow allowed for differentiation between males and females, even when the overall PDFF is not significantly different, which may indicate that there is detailed and subtle textural information in PDFF maps beyond PDFF. There was also a significant association between the texture feature Variance(global) of vertebral bone marrow and paraspinal muscles, which may indicate a close interrelationship between vertebral bone marrow with the paraspinal muscles beyond a spatial and functional association.

## Figures and Tables

**Table 1:** Subject characteristics (age and BMI), PDFF and analyzed texture features for erector spinae (ES) and psoas (PS) muscles as well as the lumbar spine (LS) for all subjects (male, n = 13; female, n = 31). The *p*-values are the result of logistic regression analysis comparing the measurements of male and female subjects, adjusted for BMI and age; significant *p*-values (for an alpha level <5%) are indicated by an asterisk.

	Male	Female	*p*-Value
Age [years]	54.79 ± 29.53	38.74 ± 16.73	0.043 *
BMI [kg/m^2^]	26.94 ± 4.62	24.87 ± 4.12	0.203
PDFF_ES_ [%]	7.79 ± 6.88	16.11 ± 9.27	0.014 *
Variance(global)_ES_	139.12 ± 14.36	117.65 ± 13.15	0.005 *
Skewness(global)_ES_	−0.13 ± 0.82	0.59 ± 0.55	0.016 *
Kurtosis(global)_ES_	3.25 ± 0.92	2.06 ± 1.54	0.012 *
Energy_ES_ [×10^3^]	0.98 ± 0.27	0.77 ± 0.43	0.011 *
Contrast_ES_	341.29 ± 57.20	405.40 ± 87.81	0.005 *
Entropy_ES_	11.20 ± 0.50	11.81 ± 0.74	0.016 *
Homogeneity_ES_	0.22 ± 0.02	0.20 ± 0.03	0.004 *
Correlation_ES_	0.45 ± 0.12	0.60 ± 0.14	0.010 *
SumAverage_ES_ [×10^2^]	0.22 ± 0.02	0.21 ± 0.02	0.853
Variance_ES_ [×10^2^]	0.82 ± 0.27	1.53 ± 0.98	0.012 *
Dissimilarity_ES_	10.84 ± 1.34	12.52 ± 1.99	0.005 *
PDFF_PS_ [%]	3.63 ± 5.38	5.39 ± 4.17	0.043 *
Variance(global)_PS_	95.68 ± 15.64	66.29 ± 8.62	0.002 *
Skewness(global)_PS_	−0.60 ± 0.29	−0.42 ± 0.52	0.031 *
Kurtosis(global)_PS_	0.89 ± 0.47	1.46 ± 0.82	0.042 *
Energy_PS_ [×10^3^]	0.39 ± 0.07	0.44 ± 0.11	0.151
Contrast_PS_	376.88 ± 49.26	389.69 ± 71.36	0.900
Entropy_PS_	12.11 ± 0.22	11.98 ± 0.31	0.216
Homogeneity_PS_	0.17 ± 0.01	0.18 ± 0.01	0.135
Correlation_PS_	0.51 ± 0.09	0.49 ± 0.11	0.547
SumAverage_PS_ [×10^2^]	0.25 ± 0.02	0.24 ± 0.03	0.252
Variance_PS_ [×10^2^]	0.98 ± 0.14	0.97 ± 0.18	0.686
Dissimilarity_PS_	13.12 ± 0.97	12.86 ± 1.03	0.376
PDFF_LS_ [%]	33.40 ± 6.77	33.08 ± 10.30	0.917
Variance(global)_LS_	41.00 ± 4.83	35.11 ± 3.24	0.006 *
Skewness(global)_LS_	−0.38 ± 0.45	−0.06 ± 0.65	0.049 *
Kurtosis(global)_LS_	1.10 ± 0.44	1.41 ± 1.02	0.420 *
Energy_LS_ [×10^2^]	0.21 ± 0.06	0.24 ± 0.09	0.894
Contrast_LS_	93.27 ± 83.51	79.36 ± 38.61	0.876
Entropy_LS_	9.66 ± 0.48	9.55 ± 0.59	0.919
Homogeneity_LS_	0.28 ± 0.02	0.28 ± 0.04	0.642
Correlation_LS_	0.60 ± 0.08	0.61 ± 0.05	0.491
SumAverage_LS_ [×10^2^]	0.34 ± 0.07	0.34 ± 0.11	0.976
Variance_LS_ [×10^2^]	10.1 ± 0.58	11.1 ± 0.68	0.468
Dissimilarity_LS_	6.31 ± 2.04	6.03 ± 1.47	0.831

BMI, Body Mass Index; PDFF, Proton Density Fat Fraction; ES, Erector Spinae; PS, Psoas; LS, Lumbar Spine.

**Table 2:** Partial correlation analysis between PDFF and texture features of the lumbar spine and the corresponding features in the erector spinae muscle (ES) or the psoas muscle (PS) respectively, adjusted for BMI and age.

	Lumbar Spine vs. ES	Lumbar Spine vs. PS
PDFF	0.13, n.s.	0.08, n.s.
Variance(global)	0.61, ***p* < 0.001**	0.62, ***p* < 0.001**
Skewness(global)	0.30, n.s.	0.28, n.s.
Kurtosis(global)	−0.33, n.s.	−0.31, n.s.
Energy	−0.15, n.s.	−0.32, n.s.
Contrast	0.23, n.s.	0.09, n.s.
Entropy	0.03, n.s.	0.28, n.s.
Homogeneity	0.06, n.s.	−0.15, n.s.
Correlation	0.05, n.s.	−0.11, n.s.
Sum Average	−0.14, n.s.	−0.04, n.s.
Variance	0.28, n.s.	−0.09, n.s.
Dissimilarity	0.25, n.s.	−0.07, n.s.

## Data Availability

The raw data supporting the conclusions of this article will be made available by the authors without undue reservation.
